# Fibrinogen gamma-A chain precursor in CSF: a candidate biomarker for Alzheimer's disease

**DOI:** 10.1186/1471-2377-7-14

**Published:** 2007-06-12

**Authors:** Joung Wook Lee, Hong Namkoong, Hyun Kee Kim, Sanghee Kim, Dong Whi Hwang, Hae Ri Na, Seon-Ah Ha, Jae-Ryong Kim, Jin Woo Kim

**Affiliations:** 1 Department of Neurology, Bobath Memorial Hospital, Bundang-Gu, Seongnam, Gyungki-do, 463-805, Korea; 2 Molecular Genetic Laboratory, College of Medicine, The Catholic University of Korea, Seoul 137-040, Korea; 3 Department of Obstetrics and Gynecology, College of Medicine, The Catholic University of Korea, Seoul 137-040, Korea; 4 Department of Biochemistry and Molecular Biology, Aging-assoiciated Vascular Disease Research Center, College of Medicine, Yeungnam University, Daegu, Korea

## Abstract

**Background:**

Cerebrospinal fluid (CSF) may be valuable for exploring protein markers for the diagnosis of Alzheimer's disease (AD). The prospect of early detection and treatment, to slow progression, holds hope for aging populations with increased average lifespan. The aim of the present study was to investigate candidate CSF biological markers in patients with mild cognitive impairment (MCI) and AD and compare them with age-matched normal control subjects.

**Methods:**

We applied proteomics approaches to analyze CSF samples derived from 27 patients with AD, 3 subjects with MCI and 30 controls. The AD group was subdivided into three groups by clinical severity according to clinical dementia rating (CDR), a well known clinical scale for dementia.

**Results:**

We demonstrated an elevated level of fibrinogen gamma-A chain precursor protein in CSF from patients with mild cognitive impairment and AD compared to the age-matched normal subjects. Moreover, its expression was more prominent in the AD group than in the MCI and correlated with disease severity and progression. In contrast, fibrinogen gamma-A chain precursor protein was detected very low in the age-matched normal group.

**Conclusion:**

These findings suggest that the CSF level of fibrinogen gamma-A chain precursor may be a candidate biomarker for AD.

## Background

Neurodegenerative diseases are characterized by chronic, progressive, irreversible deterioration of neurological function affecting cognition and motor-sensory functions. Diagnosis of neurodegenerative disease largely depends on clinical manifestations; patients usually present to clinicians when their symptoms interfere with activities of daily living (ADL). By the time patients present with these complaints, nerve cells have already been damaged irreversibly and progression of disease may be inevitable. For this reason, there is a need for a molecular-based diagnostic marker in biologic fluids that can identify neurodegenerative disease at an early or preclinical phase; this diagnostic marker could be a disease-modifying compound targeted to drug development [1].

Biochemical changes in the brain are well reflected in the CSF which is in direct contact with the brain extracellular space. Biomarkers have been discussed as possible diagnostic tools but have not been frequently used in research on the elderly because of the general paucity of supportive scientific data. However, there is an obvious need for greater diagnostic specificity and sensitivity across many diagnoses in the elderly, as well as good targets for therapeutic trials. Of the many possible areas for the discovery of biomarkers, the field of proteomics is especially well suited for investigation of biomarkers in the CSF which contains proteins associated with brain functioning under healthy conditions as well as with several neurodegenerative diseases. Many studies have demonstrated proteins in CSF associated with neurodegenerative disease using proteomics. Of the many neurodegenerative diseases studied, Alzheimer's disease (AD) has been most frequently reported [1-6].

AD, the most common form of dementia, is the most prevalent neurodegenerative disease and affects nearly 10% of the population after 65 years of age [7]. Like other neurodegenerative diseases, the diagnosis of AD is based on clinical findings and progression of the disease together with exclusion of other causes of dementias; only the neuropathological findings confirm a definite diagnosis. Most of the diagnostic pathological characteristics include extracellular amyloid plaque and intracellular neuofibrillary tangle [8]. While some progress has been made in the search for adequate biomarkers in the elderly, in particular with AD, much more work is needed before these potential biomarkers can be reliably used in clinical practice. Previous studies infrequently have focused on staging of the clinical phase of a disease and progression with neuropsychological investigations.

The purpose of our study was to develop a novel biomarker for AD by applying proteomics to CSF samples; our data demonstrate that fibrinogen gamma-A chain precursor protein is increased in CSF samples from the patient groups with MCI and AD.

## Methods

### Patient selection and grouping

During the study period from Oct. 2004 to Sep. 2005, a total of 60 subjects were enrolled at the Bobath Memorial Hospital. We applied proteomics approaches to analyze CSF samples derived from 27 patients with AD, 3 subjects with MCI and 30 controls. There were 30 individuals (mean age = 77.7 y, ranging from 76 to 80) with clinically diagnosed neurodegenerative diseases such as 3 MCIs and 27 ADs and 30 age-matched normal control subjects (mean age = 73.2 y, ranging from 61 to 95). None of the patients had other malignancies or active pulmonary disease. CSF was obtained from 60 subjects before therapy. All patients and controls were subject to the analysis with individual consent for the study. The use of CSF samples was approved by the Ethics Committee of our institution. The diagnosis of probable AD was made according to criteria proposed by the National Institute of Neurological and Communicative Disorders and Stroke--Alzheimer's Disease and Related Disorders Association (NINCDS-ADRDA) [9] and the diagnosis of mild cognitive impairment was made by Petersen's criteria [10].

Differences in disease progression were evaluated using the clinical dementia rating (CDR) [11]. Because it is difficult to differentiate CDR 0.5 with 1 and CDR 3 with 4, and 5, we arbitrarily subdivided them into three group: group 1 (CDR 0.5 and 1), group 2 (CDR 2), group 3 (CDR 3, 4 and 5). The subjects were classified according to whether they were controls with no cognitive deficit group or they had mild cognitive impairment (MCI). The thirty controls were defined by both the absence of significant cognitive impairment and of AD or other significant neuropathology. The patients without dementia, the control group, consisted of patients with other complaints such as back pain and headache. Neuropsychological testing was performed on all participants to confirm their cognitive status.

### CSF samples

CSF was obtained by lumbar puncture from individuals. The stage of disease at the time of lumbar puncture was variable. 10 ml of CSF was obtained and aliquoted into samples of 1 ml each; then they were shipped on dry ice and stored at -70°C until needed.

### Two-dimensional gel electrophoresis

Briefly, 125 μl aliquots of CSF (containing approximately 50 μg of protein) were precipitated overnight at -20°C using ice-cold ethanol. The resulting protein pellet was dissolved in 125 μl of 7 M urea, 2 M thiourea, 4% CHAPS, 60 mM dithiothreitol, 0.5% carrier ampholytes and a trace of bromophenol blue. The sample was then hydrated directly into commercially available pH 4–7 nonlinear immobilized pH gradient (IPG) isoelectric focusing gels (Amersham Biosciences, Piscataway, NJ). The first dimension of separation, isoelectric focusing, was then performed at 20°C using the IPGphor isoelectric focusing unit (Amersham Pharmacia Biotec, CA) for a total of 75 kVh. The IPG gels were equilibrated in solutions containing dithiothreitol for reduction and then iodoacetamide for alkylation. The second dimension of separation, polyacrylamide gel electrophoresis, was then performed using 12 %T acrylamide slab gels. Next, 2 DE gels were stained with silver nitrate for mass spectrometry analysis and confirmation of proteins that existed in low abundant spots [12]. Gels were fixed overnight in 30% ethanol, 10% acetic acid and washed with 20% ethanol for 20 min. The gels were sensitized for 1 min in 0.02% sodium thiosulfate and then the gels were incubated in 0.2% silver nitrate for 45 min. Development of protein spots were in 3% potassium carbonate, 0.025% formaldehyde, 0.001% sodium thiosulfate and were scanned on a GS-800 Calibrated Densitometer (Bio-Rad Laboratories, Seoul, Korea). Gels were run in duplicate and spots were selected that appeared consistently in all of the runs.

### In-gel digestion

We excised 1 mm cutting diameter protein spots to analyze. In-gel digestion was then performed according to protocols previously described [13]. Briefly, gel plugs were destained with solution mix of 30 mM potassium ferricyanide and 100 mM sodium thiosulfate by 1:1 (v/v); the gel plugs were washed 3 times for 15 min in distilled water. Destained gel plugs were shrank by 50 mM ammonium bicarbonate, acetonitrile and then dried down in a vacuum centrifuge. The proteins were then reduced and alkylated using solutions of 10 mM dithiothreitol and 100 mM iodoacetamide. Gel plugs were washed with 50 mM ammonium bicarbonate and dried with acetonitrile which were then rehydrated with a solution of trypsin (sequencing grade; Promega, Madison, WI) (5 μg/ml). The digestion process was performed overnight at 37°C and was stopped by addition of 3% formic acid. For some low-concentration spots, two or three gel plugs were digested together to increase the peptide yield. Samples were crystallized onto a MTB AnchorChip TM 600/384 (Bruker Daltonik GmbH, Leipzig, Germany) MALDI plate using the dried droplet method. For the matrix, a solution of 20 μg/μl α-cyano-4-hydroxycinnamic acid (CHCA) in 0.1% triflouroacetic acid/ACN (v/v = 70:30) was used. CHCA matrix solution was diluted 10 times with ethanol/acetone (v/v = 70:30) and then peptides samples were diluted 10 times in CHCA matrix solution.

### MS analysis

Linear and reflectron MALDI-TOF mass spectra were acquired on an Ultraflex mass spectrometer (Bruker Daltonik). Positive ion spectra were recorded using aluminum sample holders with standard parameter; a nitrogen laser (λ = 337 nm), 39 ns pulse duration and 20 kV for accelerating voltage. Peptide mass fingerprint data were collected in the positive MS reflector mode in the range of 500–4000 mass-to-charge ratio (m/z) using 1000–3000 laser shots for each sample and were calibrated internally using trypsin autolysis peaks. The spectra were analyzed using flexAnalysis (Version 2.2, Bruker Daltonik GmbH), which acts as an interface between the BioTools (Version 2.2, Bruker Daltonik) database containing raw spectra and a local copy of the Mascot search engine [14]. The MS data were searched together against a locally stored copy of the NCBInr human protein database using the Mascot search engine. The search allowed for up to one missed trypsin cleavage site, oxidation of methionine, and carbamidomethylation of cysteine.

## Results

### Expression patterns of CSF protein spots in normal, MCI and AD

**Figure 1:**
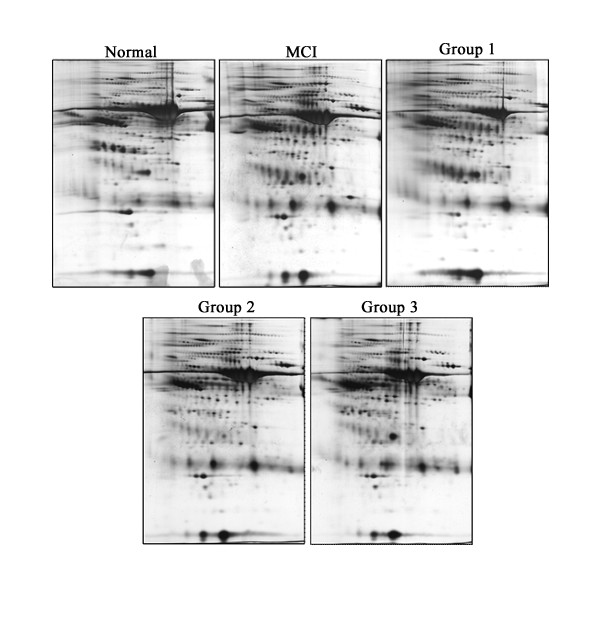
**Comparison of the 2-DE gels loaded with CSF from a AD patient**. All gels were loaded with 50 μg CSF proteins and the second dimension of separation, polyacrylamide gel electrophoresis, was then performed using 12 %T acrylamide slab gels. 2-DE gels were silver stained to visualize all proteins.

Figure 1 shows 2-DE images of gels from the CSF samples of normal control, MCI, and AD (group 1, 2, and 3 according to the CDR). On gels loaded with 50 μg of the CSF sample, 350 spots were detected. A visual comparison of spot patterns on the 2-DE gels from the CSF samples changed gradually in MCI and AD compared to normal controls. Although the individual spot intensities are variable between samples, the spot patterns are very similar. All CSF samples used in this experiment, to analyze relative differential expression, contain all proteins including albumin and IgG.

### Identification of a novel candidate protein whose expression in CSF is altered relative to normal controls

We analyzed the differential protein expression of AD patients' CSF samples, to find candidates for AD-associated proteins; selective protein identification was performed by peptide mass fingerprinting with delayed extraction-matrix assisted laser desorption/ionization-time of flight-mass spectrometry (DE-MALDI-TOF-MS). Their identification baseline was noted first, with a high Mascot score more than 67 points, and second, with a molecular weight and pI value similar to identified proteins, and finally with a high coverage from identified protein full sequence.

**Figure 2:**
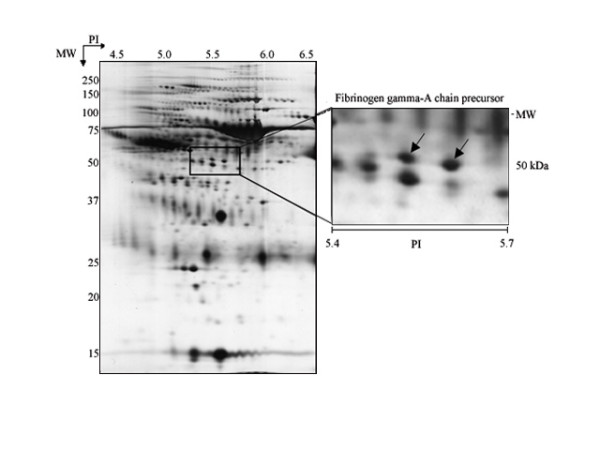
**Two-dimensional gel electrophoresis gel image using a CSF sample from an AD patient**. Spots that were found to particularly expressed spots are arrow marked. These spots identified by fibrinogen gamma-A chain precursor. The identified spots had a molecular weight of about 50 kDa and a pI of 5.6.

In total, two over-expressed protein spots were identified (Figure 2). These two spots corresponding to a molecular weight of 50 kDa were identified as a fibrinogen gamma-A chain precursor. The detailed data for the identified protein is described in Table 1.

**Table 1:** Identified CSF proteins particularly expressed in spots

Accession No.	Protein	Mascot score*	Matched peptides	Sequence coverage^†^	Protein pI	Protein MW (kDa)
P02679	Fibrinogen gamma-A chain precursor	80	20	44.9%	5.70	50.092

*Total MASCOT score: sum of individual matched peptide scores

† Percentage amino acid (AA) sequence coverage

### Fibrinogen gamma-A chain precursor

**Figure 3:**
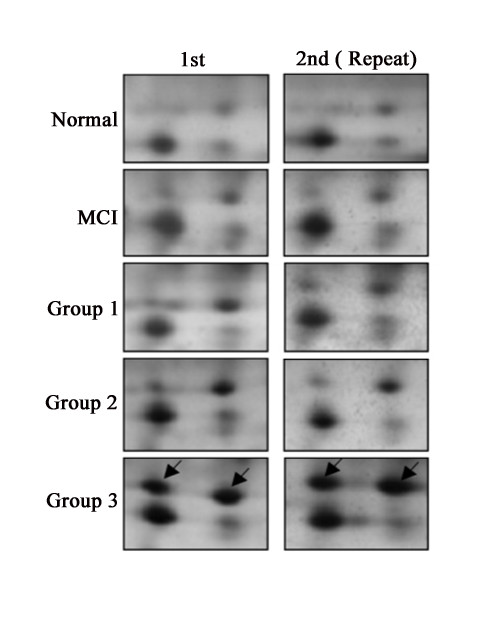
**Differentially expressed spots of fibrinogen gamma-A chain precursor**. Shown (arrow marked) is the increase in expression pattern according to progression of disease. The findings were confirmed on a repeat experiment.

Fibrinogen gamma-A chain precursor was observed to gradually increase along with the progression of disease and showed the highest expression in group 3. The clinical feature of group 3 is characterized by endstage of dementia symptoms includingseverely impaired memory and cognition, and devastated personal, whichresult in totally dependent activities of daily living. The two spots were found in all five patients and the molecular weight was about 50 kDa. A pI of 5.6 was also noted, and confirmed a very high reproducibility in a repeated experiment (Figure 3).

## Discussion

The diagnosis of AD usually relies on excluding other disorders with similar clinical features. To facilitate an early diagnosis, additional diagnostic tools related to causes of neuronal degeneration would be of great interest [15]. Such molecules that have been shown to be associated with AD are free radicals and oxidative stress promoting molecules, proinflammatory cytokines and neurotoxic agents [16]. Most of these biomarker molecules are studied in the blood and may not reflect central pathologic processes occurring in the brain of patients with AD. Therefore, CSF is a more suitable biological fluid for study of biomarker molecules in AD [17,18]. Of the many potential areas for study of CSF, the field of proteomics is especially well suited for discovery of biomarkers in CSF; this is because proteins are abundant in CSF [15,19].

Proteomics has emerged in the last few years as a multidisciplinary and technology-driven science that focuses on proteomes: the complex of proteins expressed in biological systems, their structures, interactions and post-translational modifications. In particular, proteomics examines changes in protein levels and other protein alterations that result from or foster specific diseases, or are affected by various external factors, such as toxic agents.

The combination of immunoassays and proteomic methods reveals that CSF proteins express differential protein patterns in AD, frontotemporal dementia (FTD), and PD patients; these findings suggest divergent underlying pathophysiological mechanisms and neuropathological changes underlying these diseases.

Potential biomarkers with pathophysiologic significance have been studied in the field of AD research with some success, especially in the area of genetic markers (apolipoprotein E epsilon4 allele), neuroimaging, and cerebrospinal fluid markers (Aβ42 and tau). Of these, results using proteomics combined with immunochemical studies are the most abundant. To date, combined clinical examinations and measurement of the biochemical markers (β-amyloid and tau) in CSF have become valuable diagnostic tools for predicting more than 80% of AD cases [20]. Other proteins known to be associated with AD pathology are apolipoprotein E (apo E) and synaptic proteins which have been studied by immunoassays [16]. Other candidate CSF biomarkers include: ubiquitin [21], NF protein [22], GAP43 (neuromodulin) [4,23,24], NTP, and AD7c protein [25-27]. An increasing number of studies suggest that supplementary use of these CSF markers preferably in combination, adds to the accuracy of AD diagnosis [20].

Fibrinogen gamma chain (FGG) is the gamma component of fibrinogen, a blood-borne glycoprotein comprised of three pairs of non-identical polypeptide chains. FGG A precursor is one of the transcript variant isoforms due to alternative splicing [28].

The functional features of the FGG include participation in fibrin polymerization and cross-linking, the initiation of fibrinolysis, a role in binding and regulating factor XIII activity, high affinity binding sites for integrin of platelets, leukocyte, and a role in mediating thrombin binding to fibrin, an inhibitory function originally termed 'antithrombin I' [29].

It is well known that hemostatic factors and inflammatory proteins are closely related to atherosclerosis and cardiovascular risk [30,31]. An additional hypothesis is that these factors might be related to vascular dementia. There are many reports that have discussed this relationship [32]. However, there is limited evidence, and a paucity of information, on hemostatic markers for AD. Moreover, although there are reports on the association of AD with fibrinogen in blood [33,34], there is no information of such an association in CSF.

In our study, fibrinogen gamma-A chain precursor was found to be increased in expression in both MCI and AD patients compared to normal controls. This expression was more prominent in AD patients than in both the normal controls and the MCI group, and appeared to be related to the severity of dementia. Although we cannot explain the actual relationship between FGG and AD at this point in time, these findings suggest that activated fibrinogen gamma-A chain precursor may be an important marker for the linear progression of MCI to AD and an important factor in the severity of AD. As regards the potential use for predicting AD development, a longitudinal analysis on MCI subjects is needed to verify the conversion to AD.

However, it can not be excluded that increased levels of FGG may be due to increased blood levels and/or increased permeability across the blood-CSF barrier. Both factors may influence the CSF FGG levels and thereby may cause the differences seen between AD and controls. Further studies will have to consider these putative factors to ensure specificity of the present findings.

## Conclusion

In conclusion we present that activated fibrinogen gamma-A chain precursor may be an important marker for the progression of MCI to AD and an important factor in the severity of AD. Further study is needed to confirm these findings and to improve the understanding of the relationship between FGG, mild cognitive impairment and AD.

## Abbreviations

AD, Alzheimer's disease; Apo E, apolipoprotein E; CDR, clinical dementia rating; CSF, cerebrospinal fluid; MCI, mild cognitive impairment; FTD, frontotemporal dementia

## Competing interests

The author(s) declare that they have no competing interests.

## Authors' contributions

HYK, SK, DWH, and SH performed 2-D gel electrophoresis and MALDI. JWL, HN, and HRN contributed to collecting materials. JK and JWK participated in the study design and coordination, together with drafting the manuscript. All authors read and approved the final manuscript.

## Pre-publication history

The pre-publication history for this paper can be accessed here:


